# Circ_0007534 as new emerging target in cancer: Biological functions and molecular interactions

**DOI:** 10.3389/fonc.2022.1031802

**Published:** 2022-11-24

**Authors:** Bowen Liu, Chengbin Dong, Quan Chen, Zhenhua Fan, Yu Zhang, Yifan Wu, Ting Cui, Fuquan Liu

**Affiliations:** Department of Interventional Therapy, Beijing Shijitan Hospital, Capital Medical University, Beijing, China

**Keywords:** circRNA, circ_0007534, tumor, expression, proliferation, migration

## Abstract

Circular RNA (circRNAs), an important member of the non-coding RNA (ncRNA) family, are widely expressed in a variety of biological cells. Owing to their stable structures, sequence conservations, and cell- or tissue-specific expressions, these RNA have become a popular subject of scientific research. With the development of sequencing methods, it has been revealed that circRNAs exert their biological function by sponging microRNAs (miRNAs), regulating transcription, or binding to proteins. Humans have historically been significantly impacted by various types of cancer. Studies have shown that circRNAs are abnormally expressed in various cancers and are involved in the occurrence and development of malignant tumors, such as tumor cell proliferation, migration, and invasion. As one of its star molecules, circ_0007534 is upregulated in colorectal, cervical, and pancreatic cancers; is closely related to the occurrence, development, and prognosis of tumors; and is expected to become a novel tumor marker and therapeutic target. This article briefly reviews the expression and mechanism of circ_0007534 in malignant tumors based on the domestic and foreign literature.

## Introduction

Cancer is one of the most common diseases in the 21st century and is currently the leading cause of premature death worldwide (1, 2). The worldwide incidence of cancer is expected to increase over the next 50 years; 34 million new cancers are expected to be diagnosed by 2070, doubling the estimated number of diagnoses in 2018 (3). Despite significant advances in the diagnosis and treatment of cancer, many patients often have a poor prognosis (4–6). For example, the current first-line treatment strategy for patients with metastatic cancer is chemotherapy, which aims to slow tumor progression and improve prognosis (7, 8). During the treatment process, some cancers develop resistance to antitumor drugs or their targets owing to reduced drug uptake and impaired apoptosis mechanisms during the treatment process. The rapid development of biochemical-based nanotechnology and the combination of antitumor drugs have provided a boon to such patients (9, 10). The huge amount of medical resources devoted to fighting this malignant disease has greatly increased the economic burden on both individuals and society. Consequently, exploring biomarkers for early diagnosis and early warning signs of cancer and finding new treatment methods at the molecular level requires an urgent solution, which has great social significance.

In 1958, Francis Crick proposed the “Central Dogma” (11): genetic information transfers from DNA to RNA to protein, in which RNA plays a particularly important role. Noncoding RNA (ncRNAs) are unique RNA transcripts that do not encode proteins and are widely recognized in eukaryotic genomes (12–14). ncRNAs do not constitute transcriptional “noise;” instead, they have been shown to play critical roles in regulating cellular processes and pathways in developmental and pathological settings, especially concerning cancer (15–17). According to their length and spatial structure, ncRNAs can be divided into microRNAs (miRNAs), long non-coding RNA (lncRNAs), and circular RNA (circRNAs) (18–20). With the recent exploration of ncRNA functions, the biological significance of ncRNAs has received increasing attention.

MicroRNAs are small non-coding RNAs that play essential roles in different biological processes by post-transcriptionally regulating gene expression (20, 21). For example, the gene expression of pro-apoptotic factors BCL-2-associated X protein (BAX) and CASPASE 9 in miR-181a-overexpressing cells was significantly increased, and cell viability and PGRMC1 expression in MCF-7 cells were significantly reduced, thereby inhibiting the proliferation of tumor cells (22). LncRNAs are a new class of ncRNAs that are more than 200 nucleotides in length, have no potential to encode proteins, and play the role of new master regulators in various human diseases, including cancer (23–25). Liu et al. (26) demonstrated that the lncRNA HOXA11−AS acts as an oncogene to promote the proliferation, invasion, and metastasis of hepatocellular carcinoma cells and epithelial-mesenchymal transition through the miR-506-3p/Slug axis, which provides a new therapeutic target for hepatocellular carcinoma patients.

**Figure 1:**
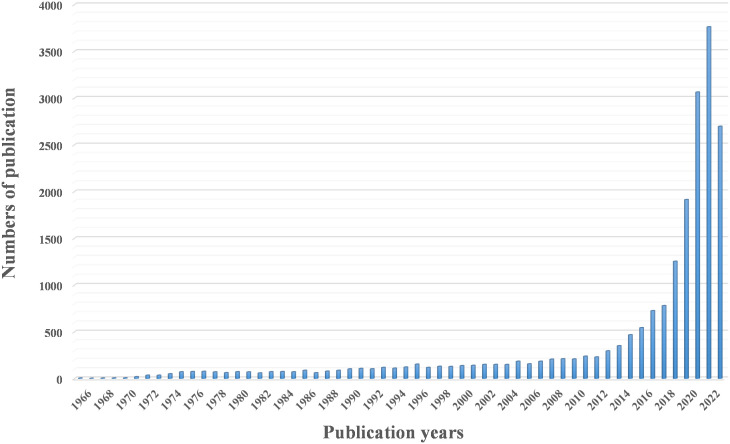
A three-dimensional cluster map of circRNA or circular RNA studies over the past decades, retrieved from PubMed.

circRNAs are a class of non-coding RNAs that are widely present in mammalian cells. They are characterized by the formation of a continuous loop without a 5’ end cap and a covalently closed 3’ end, which can be composed of exons (exonic circRNAs) or introns (intronic circRNAs) (27–29). circRNAs were first discovered in 1976 by Sanger et al. while studying viroids and have been shown to encode subviral factors (30, 31). Due to the limitations of technology and cognition at the time, circRNAs were not easily separated from other RNA species by size or electrophoretic mobility, resulting in circRNAs that were considered to be RNA molecules formed by incorrect splicing, which did not attract attention. With the rapid development of RNA sequencing technology and modern bioinformatics, an increasing number of circRNAs have been identified (**Figure 1**). It has been found that circRNA acts as an important regulatory element of the genome through various mechanisms and plays a key regulatory role in eukaryotic life activities and the occurrence and development of malignant tumors (32, 33). In contrast to linear RNAs, circRNAs have a special cyclic covalent bonding structure that makes them more resistant to exonuclease, possess the capacity to regulate gene expression and encode proteins, and thus are involved in the regulation of various diseases (34–36). Endogenous circRNA molecules act as efficient miRNA sponges, adding to the growing repertoire of regulatory functions in gene expression (37–39). Peng et al. (40) demonstrated that the deletion of circRNA_010383 promoted proteinuria and renal fibrosis in diabetic nephropathy by acting as a sponge for miR-135a. Another study (41) indicated that circACTN4 is upregulated in intrahepatic cholangiocarcinoma by acting as a molecular sponge for miR-424-5p and interacting with YBX1 to transcriptionally activate FZD7, thereby promoting the proliferation and metastasis of intrahepatic cholangiocarcinoma. A growing number of studies have shown that the expression levels of circRNAs and their patterns are significantly different between patients with cancer and their corresponding healthy controls, suggesting that various circRNA molecules in humans may represent novel biomarkers for monitoring cancer diagnosis and progression. Thus, we believe it is vital to confirm the targeted regulatory and signal transduction functions of these unusual molecules.

Among the known circRNA molecular families, circ_0007534 is a newly discovered 61869771-61877977 region located on chromosome 17, is the transcript of the DEAD-box helicase 42 (DDX42) gene, has a full length of 400nt, and is associated with tumors. In recent years, circ_0007534 has become a research hotspot in the fields of genetic information and bioinformatics. Aberrant circ_0007534 expression has been reported in many studies. At present, it has been determined that circ_0007534 expression is significantly abnormal in various tumors such as glioma, lung cancer, and osteosarcoma (**Table 1**). It is involved in malignant biological behaviors, such as tumor cell proliferation, invasion, and metastasis through various mechanisms, and the overexpression of circ_0007534 leads to a shorter survival time for patients. Therefore, we believe that with the development of bioengineering technology, circ_0007534 will provide a new perspective of RNA research as a novel tumor biomarker with high stability and high sensitivity, thereby improving the diagnosis and treatment of cancer patients.

**Table 1:** Functional characterization of circ_0007534 in various cancers.

Cancer type	Expression	Functional role	Related gene	Role	Refs.
Pancreatic cancer	Up	Proliferation, migration, invasion, apoptosis	MiR-625, miR‐892b	Oncogene	(42)
Lung cancer	Up	EMT, proliferation, migration, invasion, cell viability, clonogenic capacity	–	Oncogene	(43)
Glioma	Up	Proliferation, migration, invasion, apoptosis	MiR-761/ZIC5, miR‐22‐3p/PROX1	Oncogene	(44, 45)
Colorectal cancer	Up	Proliferation, metastasis, apoptosis	–	Oncogene	(46, 47)
Cervical cancer	Up	Proliferation, invasion, apoptosis, colony formation, viability	MiR-498/BMI-1, miR-206/GREM1	Oncogene	(48, 49)
Osteosarcoma	Up	EMT, proliferation, migration, invasion, apoptosis	AKT/GSK-3β, miR-219a-5p/SOX5	Oncogene	(50, 51)
Breast cancer	Up	Proliferation, colony formation, invasion, apoptosis	MiR-593/MUC19	Oncogene	(52)
Retinoblastoma	Up	Proliferation, cell viability, colony formation, apoptosis	MiR-214-3p, PI3K/AKT/mTOR	Oncogene	(53)
Endometrial cancer	Up	EMT, proliferation, invasion, metastasis, apoptosis, paclitaxel resistance	MiR-625/ZEB2	Oncogene	(54)

## Circ_0007534 dysregulation in cancers and its mechanisms

### Pancreatic cancer

Pancreatic cancer is the third and seventh leading cause of cancer-related deaths in the United States and worldwide, respectively (55, 56). Unlike other cancers, studies have shown that mortality from pancreatic cancer is increasing annually in European populations and is projected to be the second leading cause of cancer-related deaths in the United States by 2030 (57–59). Pancreatic ductal adenocarcinoma (PDAC) is the most common pathologic stage of pancreatic cancer, accounting for approximately 80–90% of cases (60, 61). Frustratingly, 90% of tumors are not diagnosed until they have spread beyond the pancreas at an advanced stage (42), which, combined with invasion of adjacent blood vessels, early metastasis, late diagnosis, poor treatment tolerance, and resistance to antineoplastic drugs, makes the outcome of PDAC highly unsatisfactory (61–64). Early diagnosis and treatment are key to improving the prognosis of pancreatic cancer, and adjuvant therapy, such as radiotherapy and chemotherapy after surgery, can improve the survival rate (57, 65). Globally, efforts to find effective means of early diagnosis and treatment strategies for PDAC remain challenging.

After collecting tissue samples from 60 patients with PDAC, Hao et al. (66) used a real-time quantitative polymerase chain reaction (RT-qPCR) to detect circ_0007534. In the expression of 60 PDAC tissue samples and different cell lines, it was found that the content of circ_0007534 in the cancer group was significantly higher than that in the adjacent normal tissue. After exogenous silencing of circ_0007534, tumor cell proliferation, invasion, and colony formation were inhibited. Animal experiments further clarified that downregulation of circ_0007534 could significantly inhibit tumor growth and reduce tumor weight. Bioinformatics prediction, luciferase reporting, and western blot experiments demonstrated that circ_0007534 exerted its oncogenic effects mainly through the sponge adsorption of miR-625 and miR-892b. Sample data and clinicopathological analyses revealed that positive lymph node infiltration was also correlated with the expression of circ_0007534, while sex, age, tumor location, and degree of differentiation were not significantly correlated with the expression of circ_0007534; the clinical stage and prognosis of pancreatic cancer were related to the expression of circ_0007534. In conclusion, circ_0007534 may be a novel tumor marker for pancreatic cancer diagnosis and prognosis evaluation in the future.

### Lung cancer

Lung cancer is one of the most common malignancies in the world and the leading cause of cancer-related deaths in North America and other developed countries (67–70). Lung cancer can be divided into two categories based on histology: small cell and non-small cell lung cancer (NSCLC). NSCLC is the main type, accounting for approximately 85% of all cases (42, 43, 71, 72), and is usually diagnosed at an advanced stage. The 5-year survival rate for patients treated by surgery has dropped from 90% (stage IA) to 40% (stage IIIA) because of the ineffectiveness of conventional metastatic lung cancer treatments (73). Although some potent molecular targets have been identified, they cannot be effectively utilized because of the lack of suitable drug carriers. To date, patients with metastatic NSCLC can only be treated with palliative care to improve their quality of life and prolong survival (74, 75). Consequently, the search for biomarkers with high specificity and sensitivity and the discovery of new therapeutic targets has extraordinary clinical significance for the diagnosis and treatment of lung cancer.

Qi et al. collected the pathological tissues of 98 patients with NSCLC and detected the expression of circ_0007534 in NSCLC tissue samples and non-tumor samples using RT-qPCR. The results showed that the expression of circ_0007534 in NSCLC tissues was 2.405 times higher than that in normal tissues. Compared to the normal cell line group, the expression level of circ_0007534 in NSCLC cells was significantly increased. After dividing the 98 patients with NSCLC into low- and high-expression groups, it was found that the high expression of circ_0007534 was closely related to the progression of TNM staging and positive lymph node infiltration in NSCLC patients. To further study the prognostic role of circ_0007534, Qi et al. (76) used Kaplan–Meier analysis and log-rank testing to determine the survival curve, and the results showed that NSCLC patients with elevated levels of circ_0007534 had a shorter survival time. Subsequently, univariate and multivariate Cox analyses showed that high circ_0007534 expression and advanced TNM were validated as independent prognostic predictors of 5-year overall survival in NSCLC patients. On this basis, short-strand interfering RNA (siRNAs) were constructed for subsequent experiments. The results of follow-up experiments, such as Cell Counting Kit-8 (CCK-8), Clone Formation Assay, and flow cytometry, showed that exogenous downregulation of circ_0007534 significantly inhibited the proliferation of cancer cells, impaired the ability of cancer cells to form clones, and reduced the expression of circ_0007534. The number of apoptotic cells significantly increased. Subsequent caspase-3/-9 assays yielded results similar to those obtained by flow cytometry. In addition, this study showed that circ_0007534 regulates the migration and invasion of cancer cells by affecting the epithelial-mesenchymal transition (EMT) pathway. Inhibition of circ_0007534 can significantly impair the migration ability of cancer cells, whereas upregulation of circ_0007534 produces the opposite effect. Six-week-old female BALB/c nude mice and transfected A549 cells were used for xenograft studies. Unsurprisingly, subcutaneously transplanted tumors in mice in the overexpressed circ_0007534 group grew faster and had a significantly higher tumor mass than those in the negative control group. Immunohistochemical assays also confirmed the results of *in vitro* experiments; that is, compared with the empty vector group, the expression levels of Ki67 and N-cadherin were higher, while in the circ_0007534 upregulated group, the relative expression level of E-cadherin decreased. However, the downstream mechanism and signal transduction pathway of circ_0007534 remain unknown. These findings suggest that circ_0007534, which is highly expressed in NSCLC, can regulate the malignant biological behaviors of cancer cells, such as proliferation, migration, and invasion, and is expected to become a potential therapeutic target and prognostic indicator.

### Glioma

Glioma is a common malignant tumor originating from the central nervous system, accounting for 20–30% of intracranial tumors (77, 78). They occur at all ages and are more common in adults, with males more susceptible to infection than females (44). Malignant gliomas are associated with aggressive growth, angiogenesis, necrosis, and a poor prognosis (45, 79). Patients with highly malignant gliomas have a high recurrence rate even after radiation, chemotherapy, or combination therapy, with a median survival of 15 months and a 5-year survival rate of only 5% (80–82). Therefore, the potential molecular mechanisms underlying the malignant biological behavior of glioma should be further investigated to provide targets for the diagnosis and innovative treatment of patients with glioma.

Li et al. (83) compared the expression of circ_0007534 in 35 glioma specimens and 35 normal brain tissue specimens by RT-qPCR analysis and found that circ_0007534 expression was significantly upregulated in glioma tissues, and the circ_0007534 expression level was positively correlated with glioma tumor grade (III/IV). Further experiments showed that the expression of circ_0007534 in glioma cell lines is higher than that in normal human astrocytes. This suggests that circ_0007534 overexpression may be involved in glioma progression. When exploring the regulatory mechanism of circ_0007534 in glioma, Li et al. showed that the expression of miR-761 was significantly increased after exogenous downregulation of circ_0007534, and the expression of miR-761 was significantly downregulated in glioma tissues compared to that of normal brain tissues. Bioinformatics analysis showed that ZIC family member 5 (ZIC5) is the most potential target of miR-761. RT-qPCR and western blot analyses showed that overexpression of miR-761 significantly inhibited the expression of ZIC5 protein in glioma cells. CCK-8 and transwell assays demonstrated that ZIC5 downregulation significantly inhibited the proliferation and migration of tumor cells. In conclusion, circ_0007534 acts as a glioma regulator by inhibiting miR-761 and promoting ZIC5 protein expression.

Interestingly, upon collecting and studying 45 glioma tissues from glioma patients and 23 normal brain tissues from normal controls for clinical experiments, Zheng et al. (84) also found that circ_0007534 was significantly overexpressed in cancer tissues and verified that silencing circ_0007534 inhibited cell migration and invasion. In contrast, Zheng et al. performed a cross-well invasion assay and detected caspase-3 activity using a caspase-3 kit and the apoptosis rate using flow cytometry. The experimental results showed that compared with the si-NC group, the number of glioma cells invaded by the circ_0007534 silencing group was significantly reduced, the activity of caspase-3 was significantly enhanced, and the cell apoptosis rate was significantly increased. Subsequently, they used dual-luciferase reporter gene analysis and rescue experiments to confirm the targeted interaction of circ_0007534 with miR-22-3p and the negative regulation of miR-22-3p, concluding that circ_0007534 silencing may inhibit the proliferation, migration, invasion, and apoptosis of glioma cells by upregulating the expression of miR-22-3p. StarBase database screening and follow-up experiments verified the targeted interaction between miR-22-3p and PROX1 and that the effect of circ_0007534 on silencing glioma cells was partly based on the downregulation of PROX1. The functional loss experiment suggested that PROX1 may regulate the biological behavior of glioma cells by regulating key protein levels. The results of the *in vivo* experiment showed that the tumor weight in the sh-circ_0007534 group was significantly lower than that in the sh-NC group. Compared with the sh-NC group, the expression of circ_0007534 and PROX1 proteins in the sh-circ_0007534 group was decreased, and the expression of miR-22-3p was enhanced. The expression of Ki-67, a proliferation-related marker, was detected by immunohistochemistry in the two groups, and the results showed that the expression level of Ki-67 in the sh-circ_0007534 group decreased. In conclusion, circ_0007534 may promote glioma cell proliferation, migration, invasion, inhibition of cell apoptosis, and other malignant biological behaviors through the miR-22-3p/PROX1 axis, which provides novel ideas for identifying early diagnostic and therapeutic targets of glioma.

### Colorectal cancer

Colorectal cancer (CRC), a gastrointestinal malignancy originating in the colon or rectum, is the third most common malignancy worldwide and the fourth leading cause of cancer-related death (46, 47). While screening reduces morbidity and mortality, approximately 25% of CRC patients have advanced disease at diagnosis, and nearly 25–50% of patients with early disease develop metastases (85, 86). Although knowledge of CRC risk factors, pathogenesis, and precursor lesions has advanced, the cause of the recent increase in cancer incidence remains largely unknown (85, 87, 88). Chemotherapy and targeted therapy have been widely used; however, the development of new and effective diagnostic and therapeutic strategies to improve survival outcomes has become a global necessity. The emergence of circ_0007534 has provided an extraordinary solution to this problem.

Zhang et al. (89) collected 33 pairs of CRC tissues and adjacent non-tumor tissues, detected the expression of circ_0007534by RT-qPCR, and found that circ_0007534 was significantly overexpressed in CRC tissues compared to non-tumor tissues, with an average up-regulation of 2.77 times. The CCK-8 assay results showed that knockout of circ_0007534 dramatically inhibited the proliferation of two tumor cells (SW620 and LoVo cell lines). Flow cytometry was used to further analyze whether circ_0007534 regulated the proliferation of CRC cells by altering apoptosis. The introduction of si-circ_0007534 into SW620 and LoVo cells increased the apoptosis rate. The activity of caspase-3 in the si-circ_0007534 group was significantly higher than that in the si-NC group. Furthermore, B-cell lymphoma 2 (BCL-2) is an anti-apoptotic gene, and BAX is a pro-apoptotic gene, which can be altered by increasing or decreasing the expression of BAX, which is an essential indicator of cell survival. Unsurprisingly, the BCL-2/BAX ratio in the si-circ_0007534 group was significantly lower than that in the control group. In addition, the relationship between clinicopathological parameters and the expression of circ_0007534 in CRC patients showed that the elevated expression level of circ_0007534 was associated with tumor stage and lymph node metastasis in CRC patients, whereas its expression had no significant relationship with age, sex, tumor size, location, etc. This confirmed the conclusion that circ_0007534 inhibits the proliferation of CRC cells, possibly partly by inducing apoptosis. Zhang et al. (90) also confirmed that the elevated expression level of circ_0007534 in the plasma is related to the T stage, N stage, metastatic phenotype, and poor differentiation of CRC patients, suggesting that the upregulation of circ_0007534 is inseparably associated with the invasiveness of CRC. To further confirm the reliability of blood-derived markers in the diagnosis of CRC, they performed a correlation analysis of the expression of circ_0007534 in the plasma and tumor tissues of CRC patients. The expression levels of circ_0007534 were positively correlated (r=0.505, P<0.001). Undoubtedly, they established that the plasma circ_0007534 level on the 14th day after tumor resection was significantly lower than that before surgery. Their experiments showed that circ_0007534 is relatively stable within 9 h of incubation at room temperature. However, with the extension of incubation time, its expression level was significantly lower than that in the control group. This strongly demonstrates that plasma circ_0007534 can be used as a tumor marker for CRC screening. The receiver operating characteristic (ROC) curve and area under the curve (AUC) were used to further evaluate its diagnostic value. The results showed that circ_0007534 had a sensitivity of 0.92 and a specificity of 0.522 for CRC patients. Kaplan–Meier survival curves revealed that CRC patients with high circ_0007534 expression levels had a significantly worse prognosis than patients with low circ_0007534 expression levels. In summary, circ_0007534 may be a potential tumor marker for patients with CRC. In the future, inhibiting the expression of circ_0007534 can be used to treat patients with CRC and improve their prognosis, which provides new opportunities for the diagnosis and treatment of CRC patients.

### Cervical cancer

Cervical cancer, which has one of the highest incidence and mortality rates among women worldwide, is also the most common gynecological cancer in developing countries (48, 91, 92). In most cases, cervical cancer tends to be malignant, which contributes to its high incidence and mortality rate (48, 49). The main treatment options include surgery and radiotherapy, but their efficacy is minimal, with a mortality rate of more than 85% in developing countries (93, 94). The incidence of most cancers increases with age; however, in the unscreened population, cervical cancer rates plateau at 40-45 years of age. Although a growing body of research has linked cervical cancer to female sex hormones, epidemiological studies on the relationship between estrogen and progesterone signaling and cervical cancer have been inconclusive (95, 96). Therefore, it is important to develop effective treatment options for cervical cancer.

After collecting pathological tissues and adjacent normal tissues of 45 cervical cancer patients, Rong et al. (97) used RT-qPCR analysis to quantify the expression of circ_0007534 in cervical cancer tissues and cell lines. As a result, the expression level of circ_0007534 was significantly upregulated in cervical cancer tissues, and the expression level of circ_0007534 in cervical cancer cell lines was significantly higher than that in normal cervical epithelial cell lines. Moreover, knockout of circ_0007534 restricts the ability of cervical cancer cells to proliferate and invade adjacent tissues. To explore the mechanism by which circ_0007534 regulates cervical cancer, Rong et al. conducted further experiments using dual-luciferase reporter genes and found that exogenous downregulation of circ_0007534 inhibited the proliferation and invasion of cervical cancer by upregulating miR-498 to inhibit the expression of BMI-1. These results illustrate that, in cervical cancer cells, circ_0007534 may participate in the regulation of cervical cancer by interacting with miR-498; thus, circ_0007534 and miR-498 may be potential therapeutic targets for cervical cancer. Similarly, Sun et al. (98) demonstrated that circ_0007534 is highly expressed in cervical cancer tissues and cells. SiHa and HeLa cell lines with a more obvious upregulation of circ_0007534 were selected for follow-up *in vitro* studies. Flow cytometry showed that the apoptosis rate of the si-circ_0007534 group was higher than that of the si-NC group. Western blot analysis showed that after inhibiting the expression of circ_0007534, anti-apoptotic proteins were downregulated and pro-apoptotic proteins were upregulated. In contrast to Rong et al., Sun et al. discovered an original miRNA/mRNA network in which circ_0007534 regulates cervical cancer. Through qRT-PCR, they found that overexpression of circ_0007534 significantly prevented the upregulation of miR-206 caused by miR-206 transfection and abolished the inhibitory effect of miR-206 on cell viability and colony formation. StarBase was used to predict the target region of miR-206, and it was found that the 3’ UTR of GREM1 contained the binding site of miR-206. Functional experiments demonstrated that transfection with GREM1 restored the inhibitory effect of si-circ_0007534 on SiHa and HeLa cell invasion. To investigate the role of circ_0007534 *in vivo*, a cervical cancer xenograft mouse model was established. After transfection with small interference RNA sh-circ_0007534, qRT-PCR and western blotting showed that compared with the sh-NC group, the level of GREM1 was downregulated, while the expression of miR-206 was upregulated and the tumor volume and weight were decreased in the subcutaneous tumor tissue of the sh-circ_0007534 group. In summary, these results clarify that circ_0007534, as a sponge of miR-206, upregulates GREM1 expression, promotes cervical cancer progression *in vitro* and cervical cancer tumorigenesis *in vivo*, and is expected to be a novel tumor marker for cervical cancer diagnosis and prognosis.

### Osteosarcoma

Osteosarcoma (OS) is one of the most common malignant tumors of mesenchymal origin. It is aggressive and mainly occurs in children and adolescents (50, 99, 100). Significant advances were made in the 1970s and the 1980s, but treatment and outcomes have remained unchanged, with an estimated 800–900 cases diagnosed each year in the United States (51, 101–103). Approximately 10–15% of patients with newly diagnosed osteosarcoma have metastatic disease, primarily in the lungs. The 5-year survival rate is approximately 60% in patients with limited osteosarcoma but only 20% in patients with metastatic or recurrent disease (100, 104). The current standard of care for OS includes neoadjuvant chemotherapy, surgical resection of the primary tumor, and adjuvant chemotherapy. However, the prognosis remains poor for most patients with metastatic or recurrent OS (105). A clinical study of 1,702 patients with OS implied that approximately 90% of OS patient deaths were due to progressive OS, with the majority of remaining deaths thought to be treatment-related (106). Therefore, it is imperative to identify suitable tumor markers, clarify the underlying pathogenesis of OS, and develop more effective diagnostic and treatment strategies.

After collecting pathological tissues from 57 patients with OS, Li et al. (52) analyzed them by qRT-PCR and found that the average transcription level of circ_0007534 in OS specimens was higher than that in normal adjacent tissues, and the average expression of circ_0007534 in OS specimens was 2.74 times that in normal specimens. To evaluate the clinical significance of circ_0007534 and the relationship between circ_0007534 and the clinical data of OS patients, Li et al. divided circ_0007534 expression in various tissues into two groups: a low expression group and a high expression group. Fisher’s exact test showed that the expression of circ_0007534 was significantly correlated with tumor size and degree of differentiation; however, its expression level was not significantly correlated with clinicopathological characteristics such as sex, age, WHO grade, and lung metastasis. Kaplan–Meier analysis and log-rank test showed that OS patients with high circ_0007534 expression had an overall survival rate of 12.90%, which was significantly lower than that of patients with low circ_0007534 expression (38.46%). Subsequently, a Cox risk model was constructed to further identify the expression of circ_0007534 and lung metastasis as independent prognostic indicators for OS in patients after surgery. The CCK-8 proliferation assay showed that the viability of U2OS and MG63 cells in the circ_0007534 silencing group was significantly lower than that in the si-NC group. Clonal formation experiments revealed that the cloning ability of the si-hsa_circ_0007534 group was significantly inhibited. These results demonstrated that the reduction of circ_0007534 inhibited the proliferation of OS cells. The number of early (Q3) and late (Q2) apoptotic cells significantly increased after hsa_circ_0007534 knockdown. This result was also verified in xenotransplantation experiments. The tumor volume and mass in nude mice in the circ_0007534 low expression group were significantly lower than those in the control group. They used western blotting and other methods to determine the molecular pathway mechanism leading to these effects. The test results showed that after circ_0007534 was silenced, the expression of p-AKT was reduced. Furthermore, the expression of p-GSK-3β was consistent with that of p-AKT, which confirmed that circ_0007534 is influenced by the AKT/GSK-3β cell apoptosis signaling pathway to exert its oncogenic effect. Collectively, circ_0007534 may be a plausible predictive marker and a therapeutic target for OS. Similarly, Zhang et al. (107) put forward their own opinions. While we found that circ_0007534 was highly expressed in OS tissues, the expression level of circ_0007534 in OS patients with stage III–IV tumors was higher than that in patients with stage I–II tumors, and the expression level of circ_0007534 in patients with lymph node metastasis was significantly higher than that in patients with negative lymph node metastasis. More importantly, Zhang et al. constructed a gene expression vector, and subsequent experiments showed that circ_0007534 upregulated the expression of SOX5 through sponge adsorption of miR-219a-5p to promote the proliferation, migration, and invasion of OS cells. Overall, targeting the circ_0007534/miR-219a-5p axis may be a potential therapeutic and diagnostic strategy for OS treatment.

### Breast cancer

Breast cancer is the most common malignant tumor in women worldwide, accounting for 25% of all cancer cases and 15.5% of cancer-related deaths in women (108, 109). In 2020, breast cancer became the most commonly diagnosed malignancy, surpassing lung cancer for the first time (110, 111). Mortality from infectious diseases, maternal and perinatal conditions, and malnutrition is declining and the 5-year survival rate after breast cancer diagnosis is more than 90% in high-income countries; however, this figure is significantly lower in many low- and middle-income countries (112). Although treatment methods for breast cancer have developed rapidly, such as surgery and targeted therapy, literature studies (53, 113) have shown that statins can improve the overall survival and breast cancer-specific survival (BCSS) of female non-metastatic triple-negative patients. However, it is of extraordinary clinical significance to explore the molecular mechanisms underlying the occurrence and development of breast cancer and to identify early warning markers for diagnosis.

Song et al. (114) collected 40 samples of breast cancer and adjacent normal tissues. qRT-PCR showed that si-circ_0007534#1 had the highest knockout efficiency, which was comparable to that of adjacent normal tissues and MCF-10A human breast epithelial cells. The expression of circ_0007534#1 was significantly upregulated in breast cancer tissues and the breast cancer cell lines MCF-7 and MDA-MB-231. In addition, compared with si-NC, MCF-7 and MDA-MB-231 cells transfected with si-circ_0007534#1 had significantly lower viability, a significantly reduced number of colonies, significantly weakened invasive ability, and a higher rate of apoptosis. Knockout of circ_0007534 resulted in significant upregulation in the G1 phase and significant downregulation in the S and G2/M phases. These results suggest that circ_0007534 may act as an oncogene of breast cancer. Screening using online tools such as CircInteractome revealed that miR-593 may be a potential target of circ_0007534. The interaction between circ_0007534 and miR593 was further confirmed by RNA precipitation and circ_0007534-specific probes. Functional experiments showed that the miR-593 inhibitor significantly improved breast cancer cell viability and invasion. Western blotting and immunohistochemical results showed that, compared with normal cells, the mRNA and protein expression levels of mucin 19 (MUC19) were significantly upregulated in breast cancer cell lines, and the expression level of MUC19 in breast cancer tissues was significantly higher than that in adjacent normal tissues. Further exploration of the mechanism of circ_0007534 regulation of cell proliferation revealed that upregulation of circ_0007534 promoted breast cancer cell proliferation and invasion through MUC19 by downregulating the expression of miR-593. Song et al. conducted a statistical analysis and found that the overall survival rate of patients with high MUC19 expression was poor, and the expression of MUC19 was positively correlated with the FIGO clinical stage. These results suggest that circ_0007534 promotes the expression of MUC19 by sponging miR-593 in breast cancer, thereby regulating malignant biological behaviors such as cancer cell proliferation and invasion.

### Retinoblastoma

Retinoblastoma (RB) is a rare retinal cancer in children, with an incidence of approximately 1/17,000 in newborns. Accounting for 3–4% of all pediatric malignancies, it is the most common intraocular malignancy in children (115–117). It is critical to identify high-risk pathological features because the presence of high-risk RB puts approximately 24% of children at risk of developing systemic metastases (118). Current data from European countries suggest that estimates of RB incidence are higher than those reported previously (54, 119, 120). RB originates from mature cone precursors in the developing retina. The primary goal of RB treatment is to save children’s lives first and foremost through early detection, treatment of eye tumors, and prevention of metastatic spread. The secondary goals are eye protection and maximization of visual potential. Although arterial and conventional chemotherapy have become effective methods for the treatment of advanced intraocular RB, side effects are inevitable and threaten health (121). Accordingly, the search for new treatment methods requires an urgent solution and is the key to ultimately conquering RB. Furthermore, recent studies have shown that circ_0007534 stands out among the other circRNAs.

Lv et al. (122) collected 40 pairs of RB and paired non-tumor tissues and found that osthole reduced the survival rate of Y-79 cells (RB cells) by preliminary analysis. The IC50 value doubled as the duration of osthole treatment increased from 24 to 48 h. The results of the colony formation assay showed that the proliferation of Y-79 cells decreased significantly with increasing osthole concentration (0, 50, 100, and 150 LM). The effect of osthole on apoptosis was assessed by flow cytometry, which showed that osthole treatment increased the apoptosis rate of Y-79 cells. Osthole significantly decreased the expression of Ki67, proliferating cell nuclear antigen and c-Myc. In terms of apoptosis-related proteins, BAX and cleaved caspase 3 levels were elevated after osthole treatment. In contrast, the BCL-2 levels in the osthole-treated group were lower than those in the control group. These results indicate that osthole inhibited Y-79 cell viability, proliferation, colony formation, and induced apoptosis. Lv et al. treated cells with osthole, osthole 740Y-P, and control groups to examine the effect of osthole on the downstream pathway mechanisms. The results showed that the ratios of p-PI3K/PI3K, PACK/AKT, and p-mTOR/mTOR in the osthole group were lower than those in the control group (P<0.01). The cyclic properties of circ_0007534 were investigated using RNase R and actinomycin D methods. RNase R analysis revealed that circ_0007534 was resistant to RNase R, whereas linear DDX42 mRNA was digested by RNase R. Cell percentage analysis showed that circ_0007534 was mainly localized in the cytoplasm and was also present in the nucleus, indicating that circ_0007534 is a circulating stable transcript and is upregulated in RB. Subsequent qRT-PCR analysis confirmed that the expression of circ_0007534 was significantly upregulated in RB tumor tissues and cells and, unsurprisingly, osthole treatment decreased the expression of circ_0007534. Subsequent bioinformatics analysis and dual-luciferase reporter assays demonstrated that osthole inhibits the PI3K/AKT/mTOR pathway through the circ_0007534/miR-214-3p axis, thereby exerting its anti-tumor effect. In addition, consistent with the *in vitro* results, further determination of the effect of circ_0007534 on RB progression *in vivo* revealed that osthole decreased the expression of Ki67 and increased the expression of cleaved caspase 3 *in vivo*. Likewise, osthole significantly inhibited tumor growth in an RB xenograft mouse model. In conclusion, osthole exerts an anti-retinoblastoma effect by regulating the circ_0007534/miR-214-3p axis and inhibiting the PI3K/AKT/mTOR pathway, which provides a novel approach for the diagnosis and treatment of retinoblastoma based on biological markers.

### Endometrial cancer

Endometrial cancer, an epithelial tumor that occurs in the endometrium of the uterine body, is the sixth most common cancer in women and its incidence is increasing worldwide (123, 124). Diagnostic incidence reached approximately 417,000 new cases worldwide in 2020, varying by region. The incidence of endometrial cancer is highest in North America (86.6/100,000), followed by eastern Europe (52.5/100,000) and central Europe (21.9/100,000) (124, 125). In less-developed countries, risk factors are uncommon, resulting in lower morbidity and higher specific mortality (123, 126). A multiethnic cohort study (127) showed that the risk of endometrial cancer varies by race or ethnicity; compared with Caucasians, Native Hawaiians have a higher risk, and Japanese-Americans and Latinos have a lower risk. BMI in adults and youth is a strong risk factor for endometrial cancer in racial/ethnic groups. Surgery is the mainstay of treatment for a vast majority of endometrial cancers (128, 129). Molecular signatures should also be incorporated into the treatment of endometrial cancer (130). Nevertheless, management of advanced, metastatic, and recurrent endometrial cancer remains challenging. Therefore, it is imperative to identify the underlying mechanisms of endometrial cancer to develop more effective treatment strategies.

A recent study by Li et al. (131) reported that the level of circ_0007534 in endometrial cancer tissues was significantly higher than that in normal tissues, and high expression of circ_0007534 was detected, especially in patients with poor tumor differentiation, advanced pathological stage, deep invasion, and cancer metastasis. The same conclusion was drawn for the cell lines. After dividing the expression of circ_0007534 into two groups according to the median, Kaplan–Meier curve analysis was performed, which revealed that the survival time of patients with high circ_0007534 expression was greatly shortened. CCK-8 assay showed that the proliferation of endometrial cancer cells was significantly reduced after circ_0007534 knockdown. The xenograft tumor model demonstrated that the growth rate of xenograft tumors in the sh-circ_0007534 group was much lower than that in the control group. This indicated that silencing of circ_0007534 suppressed the tumorigenicity of endometrial cancer cells. Detection of EMT-related genes using real-time PCR and western blot analyses showed that the expression of E-cadherin was increased, whereas the levels of vimentin, Twist1, and MMP2 were suppressed in cells silenced by circ_0007534. Transwell invasion and Caspase-Glo 3/7 assays showed that the invasive ability of cancer cells was dramatically reduced when circ_0007534 was knocked out, and caspase-3/7 activity was significantly higher than that in control cells. Furthermore, knockout of circ_0007534 improved the sensitivity of tumor cells to the chemotherapeutic drug paclitaxel, whereas overexpression of circ_0007534 significantly enhanced the resistance of endometrial cancer cells to paclitaxel. These results suggest that circ_0007534 promotes EMT and inhibits the sensitivity of endometrial cancer cells to paclitaxel. In addition, circ_0007534 was found to competitively bind to miR-625, and knockdown of miR-625 reversed the inhibitory effect of circ_0007534 silencing on cell proliferation and invasion. ZEB2 was selected for follow-up studies by querying a bioinformatics database. Western blot and CCK-8 experiments implied that overexpression of miR-625 or siRNA-induced ZEB2 knockdown sensitized endometrial cancer cells to paclitaxel, whereas inhibition of miR-625 expression or forced overexpression of ZEB2 significantly increased the resistance of endometrial cancer cells to paclitaxel. When endometrial cancer cells were transfected with siRNA against ZEB2, the level of ZEB2 was successfully decreased, the level of E-cadherin was increased, and the levels of Twist1, vimentin, and MMP2 were decreased, whereas the overexpression group produced the opposite effect. In conclusion, circ_0007534 promotes EMT, invasion, and paclitaxel resistance in endometrial cancer *via* the miR625/ZEB2 pathway.

## Conclusion and perspectives

Adversities in early screening and diagnosis, limited benefit from treatment modalities, and low survival rates are common challenges facing cancer patients worldwide. The ability to screen for biological markers and determine the surveillance and response of the treatment system while maintaining patient safety is critical. In the past few decades, the development of high-throughput sequencing technologies and the advent of RNA sequencing has made circRNAs a new hotspot in modern genetic research. CircRNA, a newly discovered special non-coding RNA, plays a crucial role in gene regulation at different levels, including the control of miRNA and protein functions, owing to its high stability, conservation, and tissue-specific expression. Consequently, it has unique properties and cytological functions in the occurrence and development of tumors. Recent data suggest that circRNAs play a role in various pathophysiologies, including B-cell activity in multiple sclerosis and fatty acid metabolism disorders in non-alcoholic fatty liver disease (132, 133).

**Figure 2:**
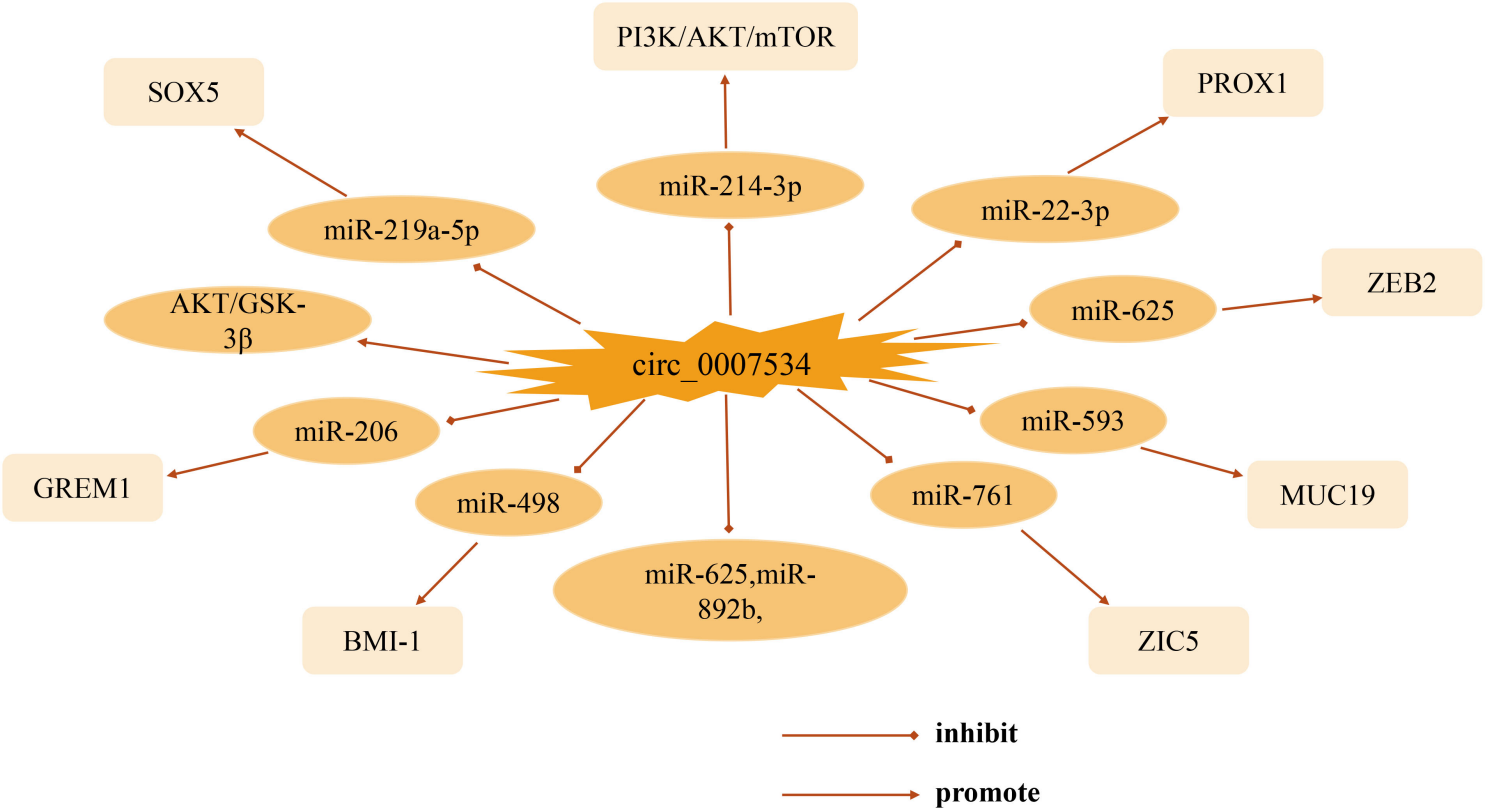
Mechanisms of circ_0007534 regulating various cancers.

This review focuses on the biological functions and molecular interactions of circ_0007534 in cancer. As one of the most prominent circRNA family members, circ_0007534 acts as a pro-cancer factor in the development of various tumors such as pancreatic cancer, glioma, non-small cell lung cancer, and breast cancer, with different mechanisms and targets of action (**Figure 2**). Thus far, significant progress has been made in understanding circ_0007534. In studies of patient clinical aspects, abnormal expression of circ_0007534 is closely related to patient clinicopathological characteristics (e.g., tumor stage, lymph node, and metastasis) and can independently predict the prognosis of patients with lung cancer, glioma, and colorectal cancer. In terms of function, circ_0007534 is closely related to tumor cell appreciation, migration, invasion, and apoptosis through multiple signaling pathways, EMT, and other mechanisms. However, in general, the in-depth study of circ_0007534 for tumors is still in its infancy, and most of these studies have focused on its ceRNA mechanism and sponge adsorption. Its resistance to chemotherapy has only been studied in endometrial cancer; however, circ_0007534 also plays a role in other cancers. What is the biological role of circ_0007534 in other cancers? It has been shown that circRNA circDLC1 inhibits mmp1-mediated liver cancer progression by interacting with HuR, and circRNA ANAPC7 inhibits pancreatic cancer tumor growth through the PHLPP2-AKT-TGF-β signaling axis, suggesting that circRNA plays a pro- or anti-cancer role in cancer development. In the present study, circ_007534 played a role as a pro-oncogene in all the cancers discussed, which raises the question of whether circ_0007534 suppresses cancer in other cancers through different pathways; further investigation of this is needed. Circ_0007534 expression should then be detected in a broader range of disease-related clinical samples such as blood, urine, and cerebrospinal fluid. It is also possible to combine the already detected circ_0007534 with tumor-related diagnostic markers that are currently used in clinic to obtain a better diagnostic value. However, whether circ_0007534 can be successfully used as an effective biomarker for cancer diagnosis and prognosis is yet to be elucidated in terms of clinical application. Moreover, understanding of the pathways and their interaction mechanisms in the process of metabolic reprogramming between circ_0007534 and the tumor microenvironment, drug resistance, and regulation of tumor cell stemness is also urgently needed for further refinement. As researchers continue to dig deeper, we believe that the above questions will be solved individually. In summary, circ_0007534 may serve as a new molecular marker for tumor diagnosis and as a potential target for tumor therapy, providing clinicians with novel approaches for the diagnosis and treatment of tumors and other diseases.

## Author contributions

BL: article conceptualization, writing, proofreading, and manuscript preparation; CD and QC: literature compilation and analysis, manuscript refinement; ZF, YZ, YW, and TC: validation of manuscripts; FL: final review and approval. All authors have contributed to the manuscript and approved the submitted version.

## Funding

This research was supported by the National Natural Science Foundation of China (81871461), Beijing Municipal Science and Technology Commission project, Capital of Public Health Cultivation (Z171100000417031), Capital Health Research and Development of Special Fund (Key Research Projects 2018-1-2081), and Sino-German Science Foundation (GZ1517).

## Conflict of interest

The authors declare that the research was conducted in the absence of any commercial or financial relationships that could be construed as potential conflicts of interest.

## Publisher’s note

All claims expressed in this article are solely those of the authors and do not necessarily represent those of their affiliated organizations, or those of the publisher, the editors and the reviewers. Any product that may be evaluated in this article, or claim that may be made by its manufacturer, is not guaranteed or endorsed by the publisher.

## Abbreviation

BAX, BCL-2-associated X protein; CCK-8, cell counting kit-8; circRNA, circular RNA; CRC, Colorectal cancer; EMT, epithelial-mesenchymal transition; lncRNA: long non-coding RNA; miRNA, microRNA; MUC19, Mucin 19; ncRNA, non-coding RNA; NSCLC, non-small cell lung cancer; OS, osteosarcoma; PDAC, pancreatic ductal adenocarcinoma; RT-qPCR, real-time quantitative polymerase chain reaction; RB, Retinoblastoma; siRNA, short-strand interfering RNA; ZIC5, ZIC family member 5.
